# Primary Broiler Hepatocytes for Establishment of a Steatosis Model

**DOI:** 10.3390/vetsci9070316

**Published:** 2022-06-24

**Authors:** Cai Zhang, Sudan Meng, Chenxu Li, Zijun Yang, Guoyong Wang, Xueying Wang, Yanbo Ma

**Affiliations:** Henan International Joint Laboratory of Animal Welfare and Health Breeding, Henan University of Science and Technology, Luoyang 471023, China; zhangcai@haust.edu.cn (C.Z.); 210321251335@stu.haust.edu.cn (S.M.); lichenxu@hnkjdx40.wecom.work (C.L.); yangzijun@haust.edu.cn (Z.Y.); wanggy@haust.edu.cn (G.W.)

**Keywords:** broiler, fat emulsion, hepatocytes, lipid metabolism

## Abstract

**Simple Summary:**

Fatty liver hemorrhage syndrome (FLHS) in chickens is a nutritional disease caused by a metabolic disorder. It mostly occurs in caged layer hens and in broiler breeders, which causes huge losses in the poultry industry. Cultured primary hepatocytes, which closely resemble the in vivo liver cell activity and physiological gene expression, have become the standard in vitro model for studying hepatic diseases. Hepatocyte steatosis models have been successfully used to study the disease in human and other animals. Fat emulsion is high in energy and contains essential fatty acids, which provide biosynthetic materials for hepatocyte steatosis. FLHS in chickens has been studied primarily using in vivo models, but rarely with an in vitro cell model. The pathological process of FLHS in vitro in both broilers and layers were shown to be similar. In the current study, to investigate the possible mechanisms of hepatic steatosis in broilers, a steatosis model was established by incubating cultured primary broiler hepatocytes with fat emulsion. In summary, the induction condition was selected as 10% fat emulsion incubation for 48 h, and we successfully established a fatty liver degeneration model for broilers, which provides the foundation for future study of fatty liver disease.

**Abstract:**

Fatty liver hemorrhage syndrome (FLHS) in chickens is characterized by steatosis and bleeding in the liver, which has caused huge losses to the poultry industry. This study aimed to use primary cultured broiler hepatocytes to establish a steatosis model to explore the optimal conditions for inducing steatosis by incubating the cells with a fat emulsion. Primary hepatocytes were isolated from an AA broiler by a modified two-step in situ perfusion method. Hepatocytes were divided into an untreated control group and a fat emulsion group that was incubated with 2.5, 5, 10, or 20% fat emulsion for different times to determine the optimal conditions for inducing steatosis of primary hepatocytes. Incubation of the cells with 10% fat emulsion resulted in cell viability at 48 h of 67%, which was higher than the control group and met the requirements of the model. In the second experiment, steatosis was induced by incubating hepatocytes with 10% fat emulsion for 48 h. In consequence, the apoptosis rate decreased (*p* > 0.05) and the concentration of ALT (*p* < 0.001), AST (*p* < 0.01), and TG (*p* < 0.05) increased significantly; the expression level of *SREBP-*1*c* (*p* < 0.05) increased, and the expression levels of *PPARα* (*p* < 0.001), *CPT*1 (*p* < 0.001), and *CPT*2 (*p* < 0.05) were lower in the fat emulsion group than in the control group. In conclusion, the induction condition was selected as 10% fat emulsion incubation for 48 h, and we successfully established a fatty liver degeneration model for broilers.

## 1. Introduction

Fatty liver hemorrhage syndrome (FLHS) in chickens is a nutritional disease caused by a metabolic disorder. It is characterized by steatosis and varying degrees of bleeding in the liver. FLHS mostly occurs in caged layer hens during the peak period of egg production and in broiler breeders [1,2], which causes huge losses in the poultry industry. Many factors are considered to be possible causes of FLHS, but imbalanced nutrition is the major one [3], similar to nonalcoholic fatty liver disease (NAFLD) in humans [4]. NAFLD begins with insulin resistance, which is more common in birds than in mammals. Insulin resistance leads to high levels of circulating free fatty acids and excess fat accumulation in hepatocytes, resulting in fatty liver [5]. Similar to nonalcoholic fatty liver disease in humans, FLHS may begin with simple hepatic steatosis. Chickens are widely considered to be a good model not only for the study of hepatic steatosis in chickens, but also for the study of NAFLD in humans [6]. Cultured primary hepatocytes, which closely resemble in vivo liver cell activity and physiological gene expression, have become the standard in vitro model for studying hepatic diseases [7]. Hepatocyte steatosis models induced by oleic acid, sodium oleate, fat emulsion, and ethanol have been successfully used to study the disease in human and other animals [8,9,10,11]. Fat emulsion, a known steatosis inducer, is high in energy and contains essential fatty acids, which provide biosynthetic materials for hepatocyte steatosis [12,13,14]. Commercial fat emulsion does not need to be prepared, and the proportion of components is stable, which is better than other inducers of hepatocyte steatosis. FLHS in chickens has been studied primarily using in vivo models, but rarely with an in vitro cell model. The pathological process of FLHS in vitro in both broilers and layers was shown to be similar. In the current study, to investigate the possible mechanisms of hepatic steatosis in broilers, a steatosis model was established by incubating cultured primary broiler hepatocytes with fat emulsion.

## 2. Materials and Methods

This study was conducted in accordance with the Declaration of Helsinki, and approved by the Animal Ethical and Welfare Committee of Henan University of Science and Technology (approval code: 2022104).

### 2.1. Isolation and Culture of Primary Hepatocytes from Broiler Chickens

Two SPF AA broilers were purchased from Pulike Biological Engineering, Inc. (Luoyang, China). They had received standard immunizations and had no clinical symptoms during feeding. The primary hepatocytes were isolated from two AA broilers by using the modified in situ 2-step perfusion method as previously published with some modifications [15]. Briefly, perfusion solution A (10.01 mM HEPES, 140.00 mM NaCl, 6.70 mM KCl, 2.53 mM glucose, 0.64 mM EDTA, pH 7.2), solution B (28.78 mM HEPES, 140.00 mM NaCl, 6.70 mM KCl, 2.53 mM glucose, 5.00 mM Ca_2_Cl, pH 7.2), and solution C (solution B containing 0.4 g/L type IV collagenase (Thermo Fisher Science Inc., Waltham, MA, USA)), were warmed to body temperature (40.9–41.9 °C) and then perfused successively through the hepatic portal vein. When it was observed that the subcapsular liver tissue was loose and cracked, the tough tissue had become soft and lost elasticity, and the outflow of the perfusion fluid began to appear turbid, the perfusion was stopped. The animals were under general anesthesia during the collection of the liver cells. After perfusion, the liver was minced to release hepatocytes. Hepatocytes were purified by centrifugation through 30% Percoll. The viable cell count was determined using a hemocytometer and trypan blue staining, and the titer was adjusted to 1 × 10^−6^ cells/mL. The cells were inoculated in six-well plates and cultured in high-glucose Dulbecco’s modified Eagle’s medium (DMEM, Thermo Fisher Science Inc., Waltham, MA, USA) with 10% fetal bovine serum (FBS, Thermo Fisher Science Inc., Waltham, MA, USA), 1 µM bovine insulin, 1 µM dexamethasone, and 100 IU/mL streptomycin-penicillin (Sigma-Aldrich, Saint Louis, CA, USA) in a humidified incubator at 37 °C in an atmosphere containing 5% CO_2_ (Thermo Fisher Science Inc., Waltham, MA, USA). After culturing primary hepatocytes for 4 h, the DMEM with 10% FBS was replaced with DMEM with 5% FBS. Subsequently, the culture medium was replaced every 24 h with DMEM without FBS. The growth and morphology of hepatocytes was observed under an inverted microscope and photographed (Olympus, Tokyo, Japan).

### 2.2. Optimal Conditions for Hepatocyte Steatosis Model Induced by Fat Emulsion

**Figure 1:**
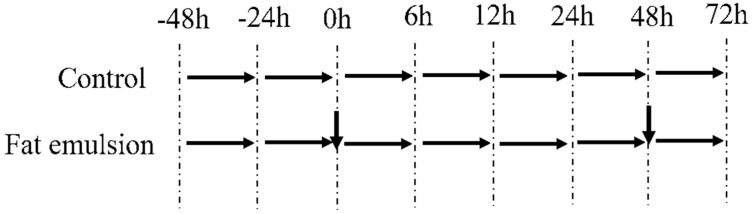
Determining optimal conditions in terms of viability for steatosis induction in primary broiler hepatocytes incubated with fat emulsion. The first downward arrow (0 h) indicates where culture medium was replaced with steatosis induction medium containing 2.5, 5, 10, or 20% fat emulsion. The second downward arrow at 48 h indicates where the medium in the fat emulsion group was replaced with fresh induction medium. Viability was determined by CCK-8 assay at 0, 6, 12, 24, 48, and 72 h.

Screening for cell viability was done by CCK-8 (Beijing Solarbio Science & Technology). The hepatocytes were seeded in 96-well plates at 5 × 10^4^ cells/well and cultured at 37 °C in 5% CO_2_ for 48 h, after which the medium was replaced with fresh DMEM + 5% FBS containing 0, 2.5, 5, 10, or 20% fat emulsion (Sichuan Kelun Pharmaceutical Co., Ltd., Sichuan, China, made up of soybean oil, medium chain triglycerides, egg lecithin, glycerol, and water for injection) for 0, 6, 12, 24, 48, or 72 h, with six replicates in each group (Figure 1). After treatment for 48 h at 37 °C, the culture medium was replaced with fresh induction medium in the fat emulsion group. The viable cell count was determined by measuring the absorbance at 450 nm with a microplate reader (Thermo Fisher Science Inc., Waltham, MA, USA) to determine the optimal conditions for the induction of steatosis. The optimum concentration and time point were used for subsequent experiments.

### 2.3. Establishing a Primary Hepatocyte Model of Steatosis in Broiler Chickens

Primary broiler hepatocytes were seeded (1 × 10^5^ cells/mL) into 6-well plates and 12-well plates (Corning, NY, USA). After culture for 24 h, cells were divided into control and fat emulsion groups with six replicates in each group. The control group received only growth medium, whereas the fat emulsion group was incubated with 10% fat emulsion. After culturing for 48 h, the cells were harvested by centrifugation and cells and supernatants were analyzed.

### 2.4. Effect of Fat Emulsion on Mitochondrial Membrane Potential in Hepatocytes

Hepatocytes were seeded into 12-well plates and treated according to the description in Section 2.2. The mitochondrial membrane potential was measured with a JC-1 kit (Nanjing Jiancheng Bioengineering Institute, Nanjing, China) according to manufacturer’s instructions. The cells were observed and imaged under a fluorescence microscope (Olympus, Tokyo, Japan). To quantify the positive area for mitochondrial membrane potential, the images were converted to binary images using the threshold function and quantified using the analyze-particle function in ImageJ (1.52a version, NIH). The ratio of green to red reflects the percentage of cells in apoptosis.

### 2.5. Effect of Fat Emulsion on Release of ALT and AST by Hepatocytes

Hepatocytes were seeded into 6-well plates and treated as described in Section 2.3. A sample (1 mL) of culture supernatant was collected from each group, and the AST and ALT concentrations were determined using commercially available kits (Nanjing Jiancheng Bioengineering Institute, Nanjing, China) according to the manufacturer’s instructions.

### 2.6. Effect of Fat Emulsion on TG Content in Hepatocytes

After fat emulsion treatment, the cells were collected and protein and TG content were determined according to kit instructions (Nanjing Jiancheng Bioengineering Institute, Nanjing, China):

Protein concentration (mg/mL) = C_standard_ × (A_sample_ − A_blank_)/(A_standard_ − A_blank_).

TG content (mmol/g prot) = [(OD_sample_ − OD_blank_)/(OD_calibration_ − OD_blank_) × calibrator concentration]/protein concentration of sample.

### 2.7. Effect of Fat Emulsion on Expression of Lipid Metabolism Genes

After fat emulsion treatment, the cells were collected and washed with precooled PBS, and total RNA was extracted using TRIzol reagent (Beijing Solarbio Technology Co., Beijing, China). RNA was reverse transcribed to cDNA and amplified by qPCR according to the kit instructions (TaKaRa Bio, Dalian, China). The concentration and purity of the RNA was determined with a NanoDrop ND-2000 spectrophotometer (Thermo Fisher Scientific, Wilmington, NC, USA) and the integrity was shown by electrophoresis of a sample of total RNA on a 1% agarose gel. RNA was transcribed into cDNA by reverse transcription using a commercial kit (Vazyme, Nanjing, China). Using *GAPDH* as the reference gene, the relative transcription levels of the lipid metabolism-related genes, *PPARα*, *SREBP-*l*c*, *ChREBP*, *CPT*1, *CPT*2, and *MTP*, were determined by quantitative fluorescence PCR using a commercial kit (TaKaRa Bio). The reference gene selected in this study was stably expressed within all samples to be compared, regardless of tissue differences, experimental conditions, or treatments. The reaction mix (20 μL) contained the following: SYBR Fast qPCR mix (10 μL), forward primer (2 μL, 2 μM), reverse primer (2 μL, 2 μM), cDNA (1 μL), and dH_2_O (5 μL). The primer sequences and amplicon sizes are shown in Table 1. All primers were synthesized by Sangon Biotech (China). The PCR conditions were as follows: 95 °C for 30 s, followed by 35 cycles of 95 °C for 10 s, 55–56 °C for 30 s, and 72 °C for 30 s, and lastly held at 4 °C. The Ct value was calculated using Excel software, and the relative expression level of mRNA was calculated using the 2^−^^ΔΔCt^ method [16], where ΔΔCt = (Ct_Experimental group target gene_ − Ct_Experimental group *GAPDH*_) − (Ct_Control group target gene_ − Ct_Control group *GAPDH*_).

### 2.8. Statistical Analysis

All experimental data were expressed as mean ± standard deviation. Differences between groups were analyzed using IBM SPSS 19.0 software (SPSS Inc., Chicago, IL, USA) and visualized using GraphPad Prism 8 (GraphPad Software Inc., San Diego, CA, USA). The statistical significance between groups was determined by Student’s *t*-test and one-way ANOVA with a Duncan test using the SPSS 19.0 software. *p* < 0.05 was defined as statistically significant. Correlation analysis (scatter plots and Pearson correlation co-efficient) was carried out using Microsoft Excel.

## 3. Results

### 3.1. Optimal Conditions for Induction of Steatosis in Broiler Primary Hepatocytes by Fat Emulsion

Fat emulsion treatment for six hours significantly promoted the proliferation of hepatocytes (*p* < 0.05) and did not affect their viability in the concentration range of 0–10% (Table 2). When the induction time with fat emulsion was >12 h, however, the cell viability in the concentration range of 0–10% gradually decreased with increasing time, and was significantly lower than at six hours for the same concentration (*p* < 0.05). With 20% fat emulsion, the cell viability decreased significantly (*p* < 0.05), possibly because the osmotic pressure of the 20% induction medium may have been so high that it damaged the cells. For the same induction time, the cell viability increased gradually with increasing fat emulsion concentration (Table 3). Cell viability was 67% when induced by 10% fat emulsion for 48 h, which could be used to establish the steatosis model using broiler primary hepatocytes.

### 3.2. Effect of Fat Emulsion on Hepatocyte Mitochondrial Membrane Potential

**Figure 2:**
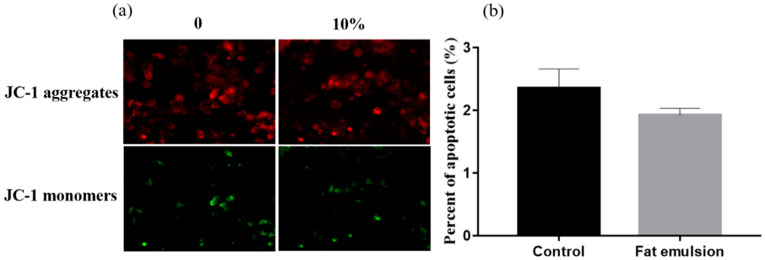
Effect of fat emulsion on mitochondrial membrane potential and apoptosis of hepatocytes. (**a**) Representative images of JC-1 staining in different groups (200×). Red fluorescence represents the mitochondrial aggregated form of JC-1, indicating normal membrane potential from intact mitochondria. Green fluorescence represents the monomeric form of JC-1, indicating loss of membrane potential (ΔΨm). (**b**) The ratio of green to red reflects the percentage of cells in apoptosis. Note: Data are presented as mean ± SEM; statistical differences were assessed by one-way ANOVA with subsequent Tukey’s HSD test (the same below).

After grouping, JC-1 staining was used to detect early apoptosis. As shown in Figure 2, a small number of JC-1 aggregates (red fluorescence) and a large number of JC-1 monomers (green fluorescence) can be seen in hepatocytes of the control group, consistent with low mitochondrial membrane potential. The green fluorescence of hepatocytes induced by 10% fat emulsion decreased, suggesting an increase in mitochondrial membrane potential (Figure 2a). Compared with the control group, the early apoptosis of cells in the fat emulsion group was lower at 48 h (*p* > 0.05), but this was not significant (Figure 2b).

### 3.3. Effect of Fat Emulsion on Release of ALT and AST from Hepatocytes

**Figure 3:**
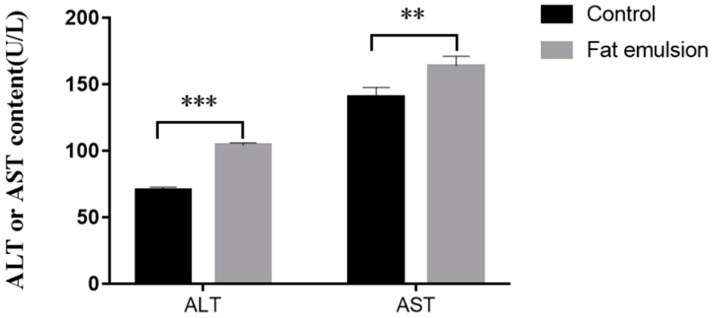
Effect of fat emulsion on the concentration of ALT and AST in the culture medium of hepatocytes. Note: Data are presented as mean ± SEM. ** *p* < 0.01, *** *p* < 0.001; statistical differences were assessed by one-way ANOVA with subsequent Tukey’s HSD test.

As shown in Figure 3, compared with the control group, the concentration of ALT (*p* < 0.001) and AST (*p* < 0.01) in cell culture supernatants of the 10% fat emulsion group was significantly higher.

### 3.4. Effect of Fat Emulsion on TG Level in Hepatocytes

**Figure 4:**
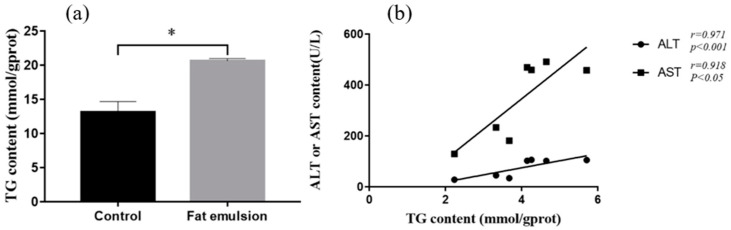
Effect of fat emulsion on TG content in hepatocytes and correlation analysis of ALT and AST with TG. (**a**) Effect of fat emulsion on TG content in hepatocytes. (**b**) The correlation analysis of ALT and AST with TG. Note: Data are presented as mean ± SEM. * *p* < 0.05; statistical differences were assessed by one-way ANOVA with subsequent Tukey’s HSD test.

As shown in Figure 4a, the TG content in the cells in the fat emulsion group was significantly higher than that in the control group (*p* < 0.05). The supernatant levels of ALT and AST were well correlated with the TG content (Figure 4b).

### 3.5. Effect of Fat Emulsion on Expression of Lipid Metabolism Genes

**Figure 5:**
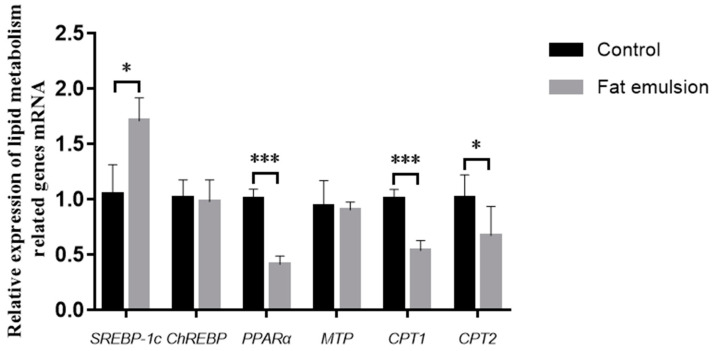
Effect of fat emulsion on the expression of lipid metabolism-related genes as determined by quantitative real-time PCR. Note: Data are presented as mean ± SEM. * *p* < 0.05, *** *p* < 0.001; statistical differences were assessed by one-way ANOVA with subsequent Tukey’s HSD test.

As shown in Figure 5, the expression of *SREBP-*1*c*, a key gene involved in lipid synthesis in cells in fat-group hepatocytes, was significantly higher than in the control (*p* < 0.05). The expression of *ChREBP*, another gene involved in lipid synthesis, was lower in the fat emulsion group than in the control group, but the difference was not significant (*p* > 0.05). The expression of the lipid metabolism gene *PPARα* in the fat emulsion group was significantly lower than that in the control group (*p* < 0.001). The expression of *MTP* in the fat emulsion group was lower than in the control, but the difference was not significant (*p* > 0.05). The expression of major genes of lipid metabolism *CPT*1 (*p* < 0.001) and *CPT*2 (*p* < 0.05) in the fat emulsion group were lower than those in the control group.

## 4. Discussion

Similar to humans, the liver is the main site of fat synthesis in broilers [4,17,18]. Although the liver can store some energy for nutritional needs, excessive nutrient levels accelerate the accumulation of fatty acids in liver tissues [13,19]. A high-energy diet during rapid growth is the main reason for FLHS in broilers [20,21,22]. There are many reports about the disease around the world, but its pathogenic mechanism is still not completely clear. The establishment of a chicken hepatocyte steatosis model provides the means for studying the pathogenesis of fatty liver disease and formulating effective prevention and treatment measures. Fat emulsion contains a large number of polyunsaturated fatty acids (PUFAs), which are easily attacked by free radicals, causing lipid peroxidation [23], which leads to fatty liver [24]. Fat emulsions also contain a lot of energy and essential fatty acids, which can provide biosynthetic materials for hepatocyte steatosis [14,25,26]. Furthermore, the excessive fatty acid exceeds the metabolic needs of the liver tissue, resulting in increased TG deposition in the liver and leading to oxidative stress in hepatocytes and fatty liver disease. Therefore, in this study we incubated primary cultures of broiler hepatocytes with fat emulsion to induce steatosis.

Most of the functions of the liver are performed by hepatocytes [27]. Studies have shown that primary hepatocytes cultured in vitro can accurately simulate the in vivo physiological environment of the liver and its activity and can be used as a model for studying drug metabolism and toxicology effects on the liver [7,28]. As highly differentiated cells, hepatocytes show a high proliferation ability in vivo, but are resistant to proliferation in vitro [29,30], where they survive for only about a week [31,32]. In this study, the primary cultured hepatocytes did not survive for more than 10 days, with viability showing a continuous decline after >60 h in culture (Table 2). Cell viability after incubation with 10% fat emulsion for >12 h decreased gradually over time (Table 2). Because of the short survival time of hepatocytes in vitro, 48 h of fat emulsion induction was selected as the condition for the establishment of the hepatocyte steatosis model. The cell viability was 67% when induced by 10% fat emulsion for 48 h, which met the requirements of modeling. Because of its high energy content and essential fatty acids, the exogenous addition of excess fat emulsion can be used to establish in vitro models of early hepatocyte steatosis. This perfectly simulates the mechanism of activation according to a “two-hit” theory [5].

ALT is mainly distributed in hepatocyte cytoplasm, whereas AST is in the mitochondria, and they are diagnostic indices of liver disease [33]. TG is a typical diagnostic indicator of fatty liver. When lipid metabolism is disordered, the content of intracellular TG will increase [24]. In the present study, the levels of ALT (*p* < 0.001), AST (*p* < 0.01), and TG (*p* < 0.05) in culture supernatants were greater in the fat emulsion group than in the control, indicating that incubation of hepatocytes with fat emulsion causes lipid metabolism disorders, leading to steatosis.

Studies have shown that many genes have important roles in lipid homeostasis [34,35]. Lipogenesis in the liver is mainly regulated by *SREBP*-1*c*, the main regulator of lipid biosynthesis [36]. *ChREBP* encodes an important transcription factor for the promotion of glycolysis and lipogenesis [37]. The decrease in fatty acid β-oxidation also leads to lipid accumulation in the liver [38]. *PPARα* is the main regulator of fatty acid oxidation, which controls expression of *CPT* and *ACADS*, two key enzymes in fatty acid β-oxidation [39]. In addition, promoting lipid transport can reduce lipid deposition. *MTP* participates in the synthesis of lipoproteins containing apolipoprotein B (apoB) and is an indispensable lipid transporter for the synthesis and secretion of very-low-density lipoprotein (VLDL) in hepatocytes [40]. In the present study, the expression level of *SREBP-*1*c* (*p* < 0.05) was greater in the fat emulsion group than in controls, whereas expression of *PPARα* (*p* < 0.001), *CPT*1 (*p* < 0.001), and *CPT*2 (*p* < 0.05) was lower in the fat emulsion group than the control group, which is consistent with earlier results [13,34,41,42]. The hepatocyte steatosis model established in this study can be used for evaluating early drug interventions.

## 5. Conclusions

In this study, primary hepatocytes were isolated from broilers by a modified two-step in situ perfusion method. This study explored the changes in activity of hepatocytes cultured in vitro. The induction condition was selected as 10% fat emulsion incubation for 48 h, and a fatty liver degeneration model for broiler chickens was successfully established. 

## Figures and Tables

**Table 1:** Primer sequences and amplicon sizes for target lipid metabolism-related genes.

Gene	Accession Number	Primer Sequence (5′→3′)	Length (bp)
*PPARα*	XM_025150258.2	F: 5′ CATTTGCTGTGGAGCTGAAGTT 3′ R: 5′ TTCCGGCATAGAATCCCACTT 3′	131 bp
*SREBP-*1*c*	XM_015294109.3	F: 5′ GAGACCATCTACAGCTCCGC 3′ R: 5′ TCCGAAAAGCACCTTCCCTC 3′	155 bp
*ChREBP*	NM_001110841.1	F: 5′ ACAGCTCAATCAACCTGTGC 3′ R: 5′ GTATGGTGGAAGGGGCAGTG 3′	184 bp
*CPT*1	NM_001012898.1	F: 5′ AACCCTTGACACAACTGGCT 3′ R: 5′ GTGACGATAAGGGCAACCCA 3′	96 bp
*CPT*2	NM_001031287.2	F: 5′ CGCGTGACGGGCCAAC 3′ R: 5′ GTTTGGGAACAGGCAGTCTGG 3′	167 bp
*MTP*	NM_001109784.2	F: 5′ TTGGCTCTCCTTTCAGGCATT3′ R: 5′ AGCCATGGATTCAGGACACC3′	225 bp
*GAPDH*	NM_204305.1	F:5′ TCGGAGTCAACGGATTTGGC3′ R: 5′ CCGTTCTCAGCCTTGACAGT3′	178 bp

**Table 2:** Effect of different concentrations of fat emulsion on hepatocyte viability at different times.

Incubation (Hours)	Fat Emulsion (%)	Cell Viability (%)	Incubation (Hours)	Fat Emulsion (%)	Cell Viability (%)
6 h	0	117.8 ± 0.046 ^ab^	48 h	0	43.6 ± 0.073 ^ghi^
	2.5	121.0 ± 0.046 ^ab^		2.5	41.8 ± 0.052 ^hi^
	5	125.0 ± 0.06 ^ab^		5	51.2 ± 0.052 ^fghi^
	10	134.4 ± 0.046 ^a^		10	66.6 ± 0.052 ^cdef^
	20	45.6 ± 0.046 ^fghi^		20	38.3 ± 0.052 ^i^
12 h	0	54.9 ± 0.046 ^efghi^	72 h	0	31.0 ± 0.073 ^i^
	2.5	73.9 ± 0.046 ^cde^		2.5	34.5 ± 0.052 ^i^
	5	87.8 ± 0.06 ^c^		5	47.3 ± 0.06 ^fghi^
	10	110.1 ± 0.046 ^b^		10	65.8 ± 0.073 ^cdefg^
	20	40.1 ± 0.06 ^hi^		20	38.3 ± 0.073 ^i^
24 h	0	44.7 ± 0.052 ^fghi^			
	2.5	62.7 ± 0.06 ^defgh^			
	5	66.9 ± 0.073 ^cdef^			
	10	77.7 ± 0.073 ^cd^			
	20	57.1 ± 0.052 ^defghi^			

Note: Different superscripts indicate significant differences between concentrations for the same incubation time (*p* < 0.0001).

**Table 3:** Effect of different concentrations and different times of fat emulsion on hepatocyte viability.

Incubation (Hours)	Fat Emulsion (%)	Cell Viability (%)
6		107.4 ± 0.345 ^a^
12		75.1 ± 0.257 ^ab^
24		59.0 ± 0.120 ^b^
48		48.8 ± 0.111 ^b^
72		42.3 ± 0.121 ^b^
	0	72.6 ± 0.3356 ^ab^
	2.5	74.8 ± 0.320 ^ab^
	5	79.8 ± 0.288 ^ab^
	10	98.0 ± 0.267 ^a^
	20	55.8 ± 0.246 ^b^
*p*-value		
Incubation time		<0.0001
Fat emulsion level		<0.0001
Interaction		<0.0001

Note: Different superscripts indicate significant differences between concentrations for the same incubation time (*p* < 0.0001).

## Data Availability

Data are contained within the article.
